# An Improved Simulated Annealing-Based Decision Model for the Hybrid Flow Shop Scheduling of Aviation Ordnance Handling

**DOI:** 10.1155/2022/1843675

**Published:** 2022-02-02

**Authors:** Xianglei Meng, Nengjian Wang, Jue Liu, Qinhui Liu

**Affiliations:** College of Mechanical and Electrical Engineering, Harbin Engineering University, Harbi 150001, China

## Abstract

Aviation ordnance handling is critical to the firepower projection of the time-critical cyclic flight operation on aircraft carriers. The complexity of the problem depends on the supply and demand features of ordnance. This paper examines the scheduling of aviation ordnance handling of an operational aircraft carrier under the framework of hybrid flow shop scheduling (HFS) and derives a method based on the simulated annealing (SA) algorithm to get the HFS problem's solution. The proposed method achieves the minimum possible flow time by optimizing the ordnance assignment through different stages. The traditional SA algorithm depends heavily on the heuristic scheme and consumes too much time to compute the optimal solution. To solve the problem, this paper improves the SA by embedding a task-based encoding method and a matrix perturbation method. The improved SA remains independent of the heuristic scheme and effectively propagates the local search process. Since the performance of SA is also influenced by its embedded parameters, orthogonal tests were carried out to carefully compare and select these parameters. Finally, different ordnance loading plans were simulated to reveal the advantage of the improved SA. The simulation results show that the improved SA (ISA) can generate better and faster solution than the traditional SA. This research provides a practical solution to stochastic HFS problems.

## 1. Introduction

This study focuses on the ordnance dispatching scheduling problem observed onboard the aircraft carrier flight operation, which plays an important role in the air wing firepower projection in its sortie generation [1]. The ordnance handling process involves many stages, equipment, and hundreds of personnel operating in a limited work space [2]; finding an optimal dispatch scheduling for a given ordnance load plan, plus the time critical nature of cyclic flight window requires, is a challenging problem. Traditionally, ordnance dispatching scheduling is made by a human operator's hand in a spreadsheet with experience, which is always nonoptimal, or even leading to delays that left aircraft launching without firepower. Thus, robust optimal scheduling is essential to conduct the ordnance handling procedure. However, such problem has seldomly been studied, which can be casted in the hybrid flow shop (HFS) scheduling framework.

The HFS scheduling problem [3] can be regarded as the combination of the flow shop scheduling (FSS) [4] problem and parallel machine scheduling (PMS) problem, where the former is to decide the job sequences through the shop and the latter is to allocate jobs to machines, given the processing times of each job on each machine according to one or several given criteria, aiming to minimize the makespan [5–7]. For an *n* jobs *m* stages problem, there are a total of (*n*!)^*m*^ possible schedules, which proves to be NP-hard [8]. If the numbers *n* of jobs and *m* of stages are very small, the optimal schedule may be determined by exhaustion, such as branch and bound (B&B) [9] or integer programming techniques [10]. However, these approaches are not applicable to HFS problems with numerous jobs and stages, due to their enormous computing time and memory occupation.

Thus, for scheduling different HFS configurations, a large number of approximation and heuristics algorithms have been proposed [11, 12]. The computational complexity of HFS propelled scholars to develop many heuristics algorithms to obtain good enough solutions in a short time for medium-to-large problems, such as different scheduling rules [13], but the heuristic methods are too problem-specific, it often cannot be applied to generalized problem. For the past decades, many general schemes on improving the performance of simple heuristics have been successfully developed, most of which are named as metaheuristics, such as genetic algorithm (GA) [14], ant colony optimization (ACO) [15], tabu search (TS) [16], neural networks (NN) [17], artificial immune systems (AIS) [18], and simulated annealing (SA) [19]. They inhere with higher level of abilities in searching the vast solution space, which have better performance than the simple heuristic methods.

Since different heuristics work effectively for different problems, when it encounters the flow shop scheduling problem, Maaroju [20] tested all the metaheuristic methods and found that the genetic algorithm and simulated annealing outperformed others, for hill climbing, swarm intelligence, and neural networks yielded only marginal improvements. However, the computation time for the GA is larger than that for SA. Thus, the SA-based algorithm is chosen in dealing with the ordnance handling problem under the HFS framework. Simulated annealing origins from the metallurgy technology, where a material cools down from high temperature to get minimum energy state. In the algorithm, the current state *s* and neighbor states *s*' are considered, and the algorithm decides the state transition probability from *s* to *s*' based on current system energy (known as temperature). This process continues until a good enough state has been found or the computation threshold has been reached. Such mechanism guarantees approximating to global optimum without getting stuck in local minimum for solving large complex optimization problems. However, the traditional SA algorithm has several defects [21], which include heuristic-dependent, parameter-specific, and long computation time; thus, the performance of the algorithm is yet to be improved. To overcome the above defects, this paper presents a Monte Carlo [22] perturbation method, which directly perturbs the solution matrix in each iteration of SA cooling, eliminating the dependence on any heuristic method, whereas SA performance also depends on cooling parameters; this paper carefully plans the calibration of these parameters to accelerate the computation process by adding double thresholds and setting the memory method of the SA.

The organizations of this paper are as follows: Section 2 introduces the ordnance handling process in detail; Section 3 discusses the methodology of the SA and improves the SA by embedding a new decoding method and matrix perturbation method; Section 4 evaluates the improved SA algorithm through computational experiments; Section 5 summarizes the research findings and gives the directions of future research.

## 2. Ordnance Handling Procedure

**Figure 1:**
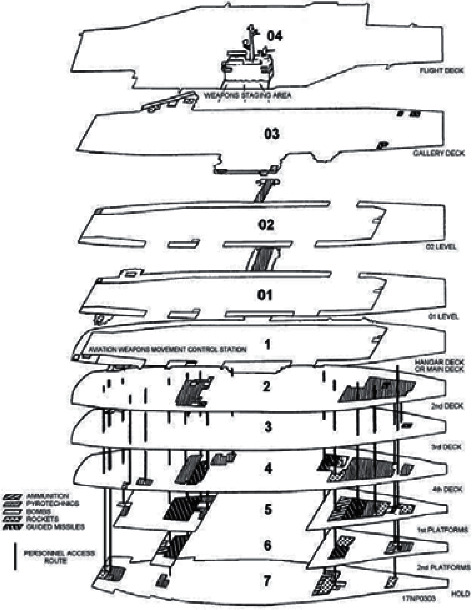
The layout of the ordnance handling routes aboard an aircraft carrier.

**Figure 2:**
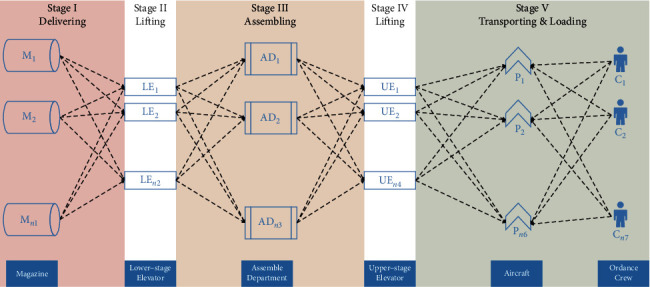
Flowchart of ordnance handling process. Note: *M* = magazine; LE = lower-stage elevator; AD = assembly department; UE = upper-stage elevator; *P* = aircraft parking spot.

The ordnance handling procedure is specified in a daily loading plan, which lists the amount and types of weapons (throughout this paper, ordnance and weapon are used interchangeably) to be loaded onto the corresponding aircraft.  Figure 1 shows the layout of aircraft carrier decks, where the construction and transfer of ordnance origin from the magazines located in lower decks to the awaiting aircraft on flight deck following a series of stages. In stage I, the ordnances are retrieved from magazines by bomb skids and delivered to lower-stage elevators. In stage II, the ordnances are lifted to the hangar deck by lower-stage elevators. In stage III, the ordnances are transferred to the staging area of the hangar deck, assembled in that area, and moved to upper-stage elevators. In stage IV, the ordnances are transferred to the flight deck by upper-stage elevators. In stage V, the ordnances are moved directly to and loaded on the aircraft waiting on the flight deck. The flowchart of this procedure is shown in Figure 2. For a common aircraft carrier layout (Figure 1), there are at least 4 magazines in the delivering stage, 8 elevators in the lower-lifting stage, 2 assemble centers in the assembling stage, 4 elevators in the upper-lifting stage, and around 10 aircraft spots in the loading stage. According to Gupta [8], the two-stage flow shop problem with one stage containing a single machine can be NP-hard. Thus, the ordnance handling problem is far from trivial, especially for ordnance officers in making timely decisions of the flight operations.

This paper examines the ordnance handling problem in the HFS framework. The definition of hybrid flow shop system is as follows: in a factory, the set of *n* jobs *J*={1,2,…, *j*,…, *n*} is going to be processed through *m* stages *M*={1,2,…, *i*,…, *m*} in sequence, while each stage *i* contains *M*_*i*_={1,2,…, *k*,…, *m*_*i*_} identical machines, and the processing time of job *j* on machine *k* is *p*_*jk*_ ≥ 0. The objective is always to minimize makespan. Similarly, in the ordnance handling problem, *n* batches of weapons are considered as jobs; they also shall be processed in the same order through *m* stages by facility *k* (as shown in Figure 2) with processing time *p*_*jk*_, and the objective is to decide the weapons' sequences and the allocations of weapons to facilities to get the minimum flow time. This is a combinatorial optimization problem with (*n*!)^*m*^ possible schedules, which is considered as NP-hard so that it is difficult to find the optimal solution in polynomial time. For a simple case of 10 batches of weapons in our problem, there can be (10!)^5^=6.3 × 10^32^ different schedules for the ordnance officer to choose, which is beyond human mind's reach in conducting the time-critical flight operations.

The ordnance configuration of aircraft on the carrier depends on the specific mission [23]. It is assumed that each transfer equipment or personnel only transfers one type of ordnance at a time [24]. For each type of ordnance, the number loaded in one skid is denoted as *r*_*w*_ Thus, the batches of ordnances needed to complete the task of all aircraft can be determined by

(1) $$\begin{array}{c} \text{task}=\left\{\begin{array}{ccccccc} 1 & \left(1,\;{\text{num}}_{1}\right) & \left(2,\;{\text{num}}_{2}\right) & ... & \left(w,\;{\text{num}}_{w}\right) & \; & \;\\ 2 & \left(1,\;{\text{num}}_{1}\right) & \left(2,\;{\text{num}}_{2}\right) & ... & \left(w,\;{\text{num}}_{w}\right) & ... & \left(W,\;{\text{num}}_{W}\right)\\ \; & \; & \; & ... & \; & \; & \;\\ a & \left(1,\;{\text{num}}_{1}\right) & \left(2,\;{\text{num}}_{2}\right) & ... & \left(w,\;{\text{num}}_{w}\right) & ... & \left(W,\;{\text{num}}_{W}\right) \end{array}\right\},\;w=1,2,\ldots W, \end{array}$$

where *a* is the number of aircraft to be loaded, *w* is the type of ordnance, and *num*_*w*_ is the actual number of ordnance types *w*.

(2) $$\begin{array}{c} \xi =\overset{a}{\underset{i=1}{\sum }}\overset{w}{\underset{j=1}{\sum }}\frac{{\text{num}}_{w}}{r_{w}},\;i=1,2,\ldots a,\;j=1,2,\ldots w, \end{array}$$

where *r*_*w*_ is the number of type *w* ordnances in one skid, *a* is the number of aircraft, and *w* is the number of ordnance types to be loaded on the aircraft. That is, an ordinance should be transferred to the required aircraft, once being retrieved from the magazine.

### 2.1. Stage I: Weapons Retrieving

Multiple magazines are located in the bow and aft of the carrier, and the ordnances can be transferred by multiple elevators. The ordnances are firstly retrieved from magazines by skids with the setup time *T*_0_. From the same magazine, the ordnances should be retrieved with an interval no shorter than *t*_int_. Then, the skids deliver the ordnances to lower-stage elevators. The time consumed to transfer ordnances from magazines to lower-stage elevators can be expressed as

(3) $$\begin{array}{c} T_{M}^{L}=\left[\begin{array}{cccc} t_{1}^{1} & t_{1}^{2} & \cdots & t_{1}^{L}\\ t_{2}^{1} & t_{2}^{2} & \cdots & t_{2}^{L}\\ \vdots & \vdots & \vdots & \vdots \\ t_{M}^{1} & t_{M}^{2} & \cdots & t_{M}^{L} \end{array}\right], \end{array}$$

where *M* is a magazine and *L* is a lower-stage elevator.

### 2.2. Stage II: Weapons Buildup

The ordnances are loaded onto lower-stage elevators and lifted vertically with constant speed to the hangar deck. The time consumed in this stage (lifting time) is denoted as *T*_*L*_.

### 2.3. Stage III: Weapons Assembling

The ordnances are preassembled in the staging area of the hangar deck, with a sufficient lead time to meet the short turnaround time of the flight schedule. The assembling time *T*_*K*_^*ass*^ varies with the types of ordnances. Note that the assembling time of the staff fluctuates in the real world. Therefore, the interval of assembling time was set to [−*T*_*f1*_, *T*_*f1*_]. The real assembling time is denoted as *T*_*K*_^*ass*^+*t*_1_, where *t*_1_ is a random number within [−*T*_*f1*_, *T*_*f1*_].

### 2.4. Stage IV: Weapons Striking Up

The ordnances are transferred to the flight deck by upper-stage elevators. The time consumed in this stage (transport time) is denoted as *T*_*U*_.

### 2.5. Stage V: Weapons Loading

Some ordnance crew members on the flight deck transport the ordnances from the upper-stage elevators to the aircraft. The time consumed in this stage can be expressed as

(4) $$\begin{array}{c} T_{U}^{A}=\left[\begin{array}{cccc} t_{1}^{1} & t_{1}^{2} & \cdots & t_{1}^{A}\\ t_{2}^{1} & t_{2}^{2} & \cdots & t_{2}^{A}\\ \vdots & \vdots & \vdots & \vdots \\ t_{U}^{1} & t_{U}^{2} & \cdots & t_{U}^{A} \end{array}\right], \end{array}$$

where *U* is an upper-stage elevator and *A* is an aircraft.

The other ordnance crew members load the ordnances onto the aircraft. It is assumed that the different groups consume the same time to load the same ordnance and different types of ordnances need different time to be loaded. The time needed to load each type of ordnance is denoted as *T*_*K*_^load^. The interval of the loading time was set as [−*T*_*f2*_, *T*_*f2*_]. The real loading time is denoted as *T*_*K*_^load^+*t*_2_, where *t*_2_ is a random number within [−*T*_*f2*_, *T*_*f2*_].

The ordnance crew members can be shared across groups. The loading cannot proceed unless ordnance crew members are available. The walking time for interstation transfer between different aircraft can be expressed as

(5) $$\begin{array}{c} T_{A}^{A}=\left[\begin{array}{cccc} t_{1}^{1} & t_{1}^{2} & \cdots & t_{1}^{A}\\ t_{2}^{1} & t_{2}^{2} & \cdots & t_{2}^{A}\\ \vdots & \vdots & \vdots & \vdots \\ t_{A}^{1} & t_{A}^{2} & \cdots & t_{A}^{A} \end{array}\right]. \end{array}$$

Referring to the standard three-field notation for scheduling problems, our problem can be described as follows: *FH*5, (*PM*^(*k*)^)_*k*_^2^=1, (*RM*^(*k*)^)_*k*=3_^4^*|prmu*, *M*_*j*_^(5)^, block*|C*_max_. Specifically, *FH*5 is a five-stage HFS problem: stage I involves *M*^(1)^ identical magazines that store ordnances; stage II involves *M*^(*2*)^ lower-stage elevators to transport the ordnances; stage III has *M*^(3)^ identical assembling personnel to assemble the ordnances; stage IV has *RM*^(*4*)^ independent upper-stage elevators to transport the ordnances; and stage V has *RM*^(5)^ independent aircraft to be loaded. Note that *prmu* indicates that the ordnances are handled in the same order in every stage; *M*_*j*_^(5)^ (eligibility constraint) means that the handling of ordnance *j* is limited to the aircraft set *M* in stage V; *block* indicates that the capacity of buffer between stages is constrained, for instance, the weapons have to wait in the current stage till enough room is released for the next stage of handling.

In total, the completion time for batch *i* of ordnances can be calculated by

(6) $$\begin{array}{c} C_{i}=T_{M}^{L}+T_{L}+T_{K}^{ass}+t_{1}+T_{U}+T_{U}^{A}+T_{A}^{A}+T_{K}^{\text{load}}+t_{2}+T_{\text{wating}},\;i=1,2,\ldots \xi , \end{array}$$

where *T*_wating_ is the whole waiting time in the transporting process, for an ordinance cannot be handled unless machines or ordnance crew members are available.

The general objective of ordnance handling is to complete all transporting operations as efficiently as possible within the specified time and to generate a reasonable schedule for ordnance handling. Therefore, for our ordnance handling problem, the minimization of makespan is set as goal, so that sufficient ordnances can be loaded to the awaiting aircraft to fly in the next flying window.

(7) $$\begin{array}{c} \text{Object}=\min\left(C_{\max}\right). \end{array}$$

## 3. Improved Simulated Annealing Algorithm

The SA is a technique capable of searching for good solutions to various combinatorial problems in material science and physics. The pseudocode of the algorithm is as follows.

The SA includes three major functions: state generation, state acceptance, and temperature update. The first is to make perturbations of the given initial solution, in order to search for the optimal solution effectively in the vast solution space. The second decides whether to accept a newly generated solution with a certain probability in case of trapping in the local minimum. The third offers a cooling scheme that mimics the physical annealing process to get a stable state for the problem. The SA performance can be augmented by adjusting various parameters and operators [25], such as initial temperature, descent gradient of temperature, and termination rule, which have to be adjusted manually. This paper mainly improves the search efficiency (timeliness) of the SA, without sacrificing the optimization quality. Thus, encoding scheme, initial solution, neighborhood search structure (NSS), type of cooling schedule, and two criteria (internal cycle termination and external cycle termination) are modified as described in the following subsections.

### 3.1. Task Encoding

This paper presents a task-based encoding method for the ordnance handling problem, where ordnances are coupled with aircraft. Each job of the HFS is defined as an operation *o*^*xy*^, where *x* is the type of ordnance and *y* is the aircraft to be loaded. Then, a solution can be expressed as

(8) $$\begin{array}{c} S=\left(\begin{array}{ccc} \begin{array}{c} o_{1}^{xy}\\ o_{2}^{xy} \end{array} & \begin{array}{c} m_{11}\ldots \\ m_{21}\ldots \end{array} & \begin{array}{c} m_{n1}\\ m_{n1} \end{array}\\ \vdots & \vdots & \vdots \\ o_{i}^{xy} & m_{n1}\cdots & m_{nn} \end{array}\right), \end{array}$$

where the first column is a permutation of the task sequence, the following columns are the corresponding stages, and *m*_1_ to *m*_*n*_ are machines assigned randomly to execute the tasks.

### 3.2. Initial Solution

As mentioned above, the initial solution has a great impact on the final solution of the SA. According to the heuristic methods mentioned in [26], we generate the initial solution by the following rule: first, the permutation of tasks is determined by assigning each type of weapon *x* to its corresponding aircraft *y*, defined as operation *o*_*i*_^*xy*^, according to the ordnance loading plan *P*. Then, the tasks are assigned to the earliest available machine. If there are more than one earliest available machines, one of them will be chosen randomly. The initial solution will be generated as *S*_*i*×*m*_ with *i* tasks through *m* stages.

For example, Table 1 shows the ordnance loading plan of the aircraft waiting to operate in the next fly window. There are three aircraft and four types of ordnances. Aircraft 1 requires 2 skids of type 1 ordnances and 1 skid of type 2 ordnances.

Here, the operations are permutated by aircraft number (equal priority) in ascending order: *o*_*i*_^*xy*^={*o*_1_^11^, *o*_2_^11^, *o*_3_^21^, *o*_4_^12^, *o*_5_^32^, *o*_6_^43^}. First, a random permutation of jobs (tasks) is generated. Then, the optimal available elevator is assigned to each operation. The final plan can be written as

(9) $$\begin{array}{c} \left(\begin{array}{cccccc} 3 & 1 & 1 & 1 & 1 & 1\\ 5 & 2 & 2 & 2 & 2 & 2\\ 1 & 3 & 3 & 1 & 3 & 3\\ 4 & 4 & 4 & 2 & 4 & 1\\ 2 & 1 & 1 & 1 & 1 & 2\\ 6 & 2 & 2 & 2 & 2 & 3 \end{array}\right). \end{array}$$

The meaning of such matrix can be explained as follows: taken the third row for illustration, operation *o*_1_^11^ is extracted out of the third magazine, transferred by the third lower-stage elevator to the first staging area, moved by the third upper-stage elevator to the flight deck, and loaded to the corresponding aircraft by the No. 3 ordnance crew.

### 3.3. Neighborhood Perturbation

The NSS can generate a new solution by slightly modifying the current candidate solution. Traditionally, many different NSSs are adopted in each iteration of SA computation, such as swap, shift, and reversion [27], to realize the task permutation in the first stage. Then, the same heuristic rules are applied in the following stages to generate a new solution S'. The quality of the solution depends on the selected heuristic rules. If the rules are too greedy, the algorithm may fall into the local optimum, being unable to get convergence to the final optimal result.

The quality of the SA solution is highly sensitive to the selection of candidate solutions. Therefore, the perturbation scheme is crucial to the good performance of the SA algorithm. To ease the dependence of neighborhood search on heuristics, this paper proposes a Monte Carlo perturbation technique, which directly perturbs the initial solution matrix. The initial solution is changed significantly in one step, eliminating the effect by heuristic methods. The matrix perturbation is described as follows.

The following is an example of the matrix perturbation process: for instance, a 6 × 3 matrix, a rectangle *R*_*a*×*b*_ randomly generated a size of 4 × 3 matrix.


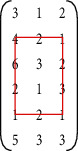
which covers the matrix of $\left(\begin{array}{ccc} 4 & 2 & 1\\ 6 & 3 & 2\\ 2 & 1 & 3\\ 1 & 2 & 1 \end{array}\right)$.

Then, matrix *R* is reversed as *R'* =  $\left(\begin{array}{ccc} 1 & 2 & 1\\ 2 & 1 & 3\\ 6 & 3 & 2\\ 4 & 2 & 1 \end{array}\right)$.

Then, the new solution can be obtained as *S*' =  $\left(\begin{array}{ccc} 3 & 1 & 2\\ 1 & 2 & 1\\ 2 & 1 & 3\\ 6 & 3 & 2\\ 4 & 2 & 1\\ 5 & 3 & 3 \end{array}\right)$.

Hence, the matrix perturbation process is completed: $\left(\begin{array}{ccc} 3 & 1 & 2\\ 4 & 2 & 1\\ 6 & 3 & 2\\ 2 & 1 & 3\\ 1 & 2 & 1\\ 5 & 3 & 3 \end{array}\right)⟶\left(\begin{array}{ccc} 3 & 1 & 2\\ 1 & 2 & 1\\ 2 & 1 & 3\\ 6 & 3 & 2\\ 4 & 2 & 1\\ 5 & 3 & 3 \end{array}\right)$.

### 3.4. Cooling Schedule

The SA behavior can be regulated by the temperature and its descent gradient. To avoid the local optimum trap, inferior solutions may be accepted depending on the falling temperature, under the mechanism of cooling schedule. Here, the exponential cooling rate is adopted:

(10) $$\begin{array}{c} T_{l}=\frac{\left(T_{0}-T_{f}\right)\left(N+1\right)}{N\left(l+1\right)}+T_{0}-\frac{\left(T_{0}-T_{f}\right)\left(N+1\right)}{N};\;l=1,2,\ldots ,N, \end{array}$$

where *T*_0_ and *T*_*f*_ are initial temperature and final temperature, respectively, and *N* is the number of temperatures between *T*_0_ and *T*_*f*_.

The SA needs to accept the new state through probability judgment, in order to avoid the local minimum. When the initial temperature is sufficiently high, the cooling is slow enough (i.e., each temperature is held for a sufficiently long time), and the final temperature approaches zero; the SA will converge to the global optimal solution with the probability of 1. However, it is very difficult to fulfil the global convergence condition. Besides, the current state may be worse than some intermediate states in the search trajectory, owing to the probability acceptance mechanism. Thus, the SA algorithm often converges to an approximate optimal solution, or a solution poorer than the best intermediate solution. The search efficiency is inevitably affected. To preserve the best-known state and improve search efficiency, this section makes the following improvements to the SA:

1. Memorize the best intermediate solution in the search process and update it immediately. The improvement of memory turns the SA into an intelligent algorithm.
2. Set up two thresholds, internal cycle threshold and external cycle threshold, to reduce the computing load while maintaining optimality. The internal cycle threshold refers to the number of cycles that the new solution of continuous disturbance does not generate a better solution at a certain temperature, while the external cycle threshold refers to the number of cycles that the new solution generated by continuous cooling does not generate a better solution. The two thresholds are determined as follows.

First, determine whether the number of internal cycles reaches the threshold; if yes, lower the temperature by one step; otherwise, judge if it conforms to Markov chain. If not, reconduct the process of state generation, state acceptance, and algorithm termination; otherwise, lower the temperature by one step. Second, determine whether the number of external cycles reaches the threshold; if yes, terminate the algorithm and obtain the final solution; otherwise, judge whether the algorithm meets the termination conditions. If yes, terminate the algorithm and obtain the final solution; otherwise, reconduct the process of state generation, state acceptance, and algorithm termination. Terminate the algorithm once the number of iterations *i* surpasses the prior fixed constant *MaxIter*. In our experiments, *MaxIter* was set to 10^4^.

**Figure 3:**
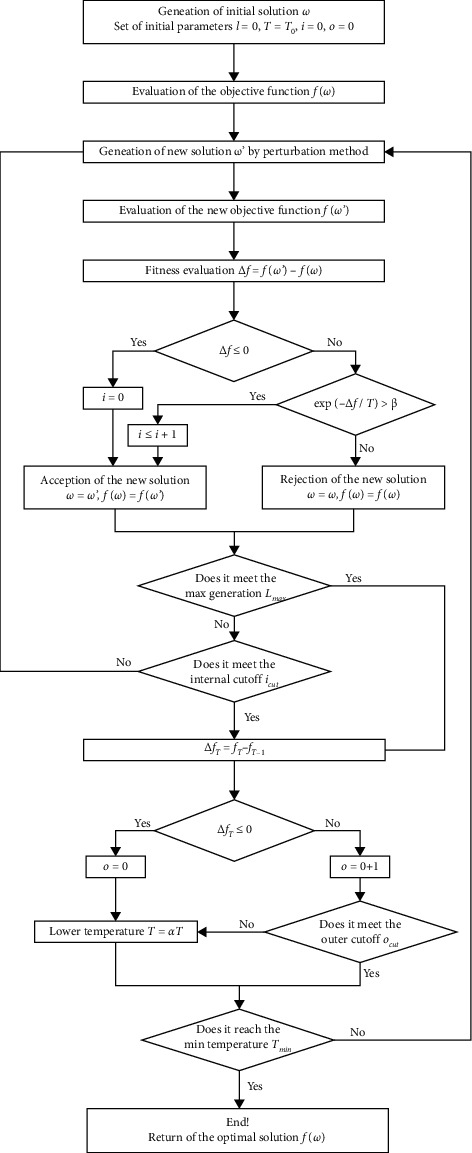
Flow of the improved SA.

The flow of the improved SA is shown in Figure 3.

## 4. Experiments

To test the effectiveness of SA-based algorithm, we first evaluate the control factors of the SA and suggest a good parameter setting. Then, the solution quality and efficiency of the ISA were verified through several experiments. The algorithms are implemented in our previously published carrier-based flight operations simulation [28], which is written by C++ and ran on Microsoft Windows operating system with 4 GB RAM and dual core CPU.

### 4.1. Parameter Tuning

The efficiency of the SA algorithm is greatly affected by the design of parameters and operators. The full factorial design tests all possible combinations. Such an approach becomes too laborious in the face of numerous factors. Taguchi utilized orthogonal arrays to examine lots of decision variables in a few tests [29] and measured the importance of each factor by its influence on algorithm performance, using the signal-to-noise ratio: 10  log  10(objective)^2^. Following Taguchi's method, the SA control factors were configured as follows: initial solution, initial temperature, cooling rate, and number of neighborhood searches in every temperature.  Table 2 shows the different levels of these factors.

Hence, the SA has one 3-level factor and three 4-level factors. The best design among the orthogonal arrays is L16. Thus, additional transform was performed to fit L16 (Table 3).

The relative percentage deviation (RPD) was also adopted to measure the performances:

(11) $$\begin{array}{c} \text{RPD}=\frac{A{\mathrm{lg}}_{\text{sol}}-{\text{Min}}_{\text{sol}}}{{\text{Min}}_{\text{sol}}}\cdot 100\%, \end{array}$$

where the best solution obtained for one instance is denoted as *Min*_sol_, while the objective value is marked as *Alg*_sol_.  Table 4 lists the S/N ratios and RPD values of each level of the factor value. The results show that A(3), B(4), C(4), and D(4) are the best levels of the factors.

### 4.2. Experimental Settings

The size of the test instances was set to *n*={15,30,45,60} tasks, which corresponds to a common mission of strike sorties of 5, 10, 15, and 20 aircraft, respectively. The processing time for jobs on each machine was generated by triangle distribution with mean time according to [30]. There are resource constraints of five types of weapons, four lower-stage elevators, 10 assembling crews, four upper-stage elevators, and six loading crew members, see Tables 5–9 for comprehensive data.

### 4.3. Makespan Analysis

The test of problem uses an ordnance loading plan, ranging from 5 to 20 aircraft. At first, the experiment tries to solve a standard small size problem with optimal solution and preliminarily demonstrates the adaptability and feasibility of the ISA. Then, the proposed ISA was adopted to solve larger size problems and compared with the other methods to reveal its superiority.

To compare the ISA with the SA, control factors were configured as those in the preceding section. The permutation of machines was arranged in ascending order. The initial temperature *T*_0_ = 10^3^, final temperature *ε* = 0.5, cooling rate *α* = 0.99, and length of Markov chain L = 2000.

**Figure 4:**
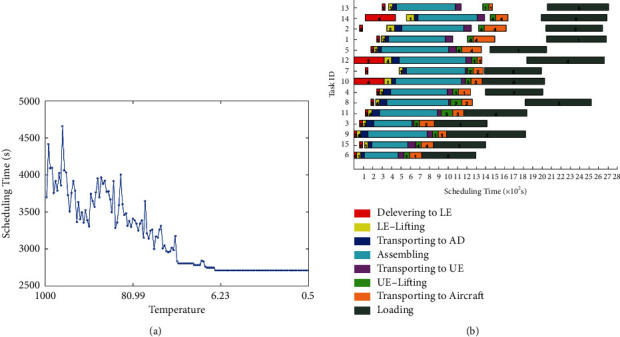
Optimal schedule of 15 tasks (5 aircraft) derived by the ISA. (a) Convergence curve. (b) Gantt chart.

**Figure 5:**
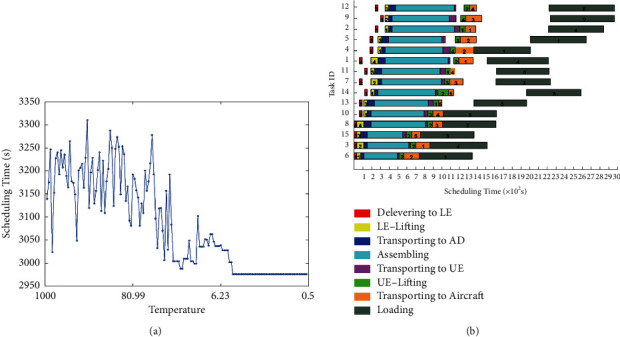
Optimal schedule of 15 tasks (5 aircraft) derived by the SA. (a) Convergence curve. (b) Gantt chart.

The base case includes 15 tasks. As the temperature declined (Figure 4(a)), the ISA converged to the optimal solution in 2.705 s, faster than the SA, which converged to the optimal solution in 2.965 s (Figure 5(a)). Although the initial scheduling time of the ISA was higher than that of SA, its faster convergence procedure suggests the good performance of the ISA. Note that the computing time of the ISA was 85.3637 s, much shorter than that (143.7861 s) of the SA.

**Figure 6:**
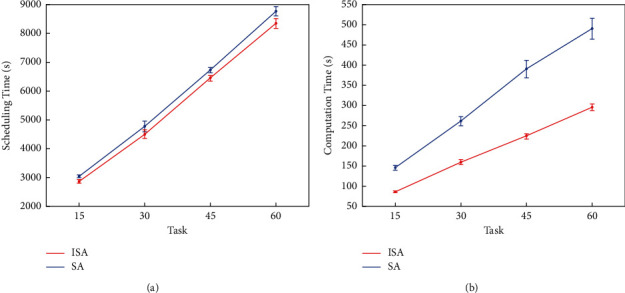
Performance of the ISA and the SA on medium and large problems. (a) Scheduling time. (b) Computing time.

Next, the ISA performance on larger problems was tested, whereas more tasks bring a greater computing effort for the heuristic.  Table 10 lists the average scheduling time and computing time of different tasks, with each task running for 30 times.  Figure 6 shows the corresponding plot. The results showed that the ISA achieved a shorter scheduling time and a faster computing speed than the SA. Comparing with the SA, the average scheduling time of the ISA reduces from 300 to 600 seconds in each ordnance turnaround cycle for 30 to 60 tasks and the average computing time saves from 110 to 190 seconds. Note that there are usually ten or more cycles in a general flight day; the scheduling time saved by implementing ISA equals one more group sortie generation, which in turn enhanced the firepower capacity of carrier air wing.

The results also show that the bottleneck of ordnance handling is the loading process, where the number of loading crews heavily influences the aircraft turnaround time. When there is high intensity of surge operations, more loading crews should be arranged to handle ordnances.

## 5. Conclusions

This paper treats the aviation ordnance scheduling problem under the HFS framework with multistages, independent parallel machines, and several processing constraints. The simulated annealing algorithm was modified with dual threshold selection to generate faster and better schedules using the proposed matrix perturbation method that keeps the SA independent of the heuristic schemes. The influencing parameters of the improved algorithm are carefully tuned by Taguchi's method. The experimental results demonstrate the effectiveness of ISA, which provides a practical solution to a broad application in dealing with stochastic hybrid flow shop scheduling problems.

## Figures and Tables

**Algorithm 1:**
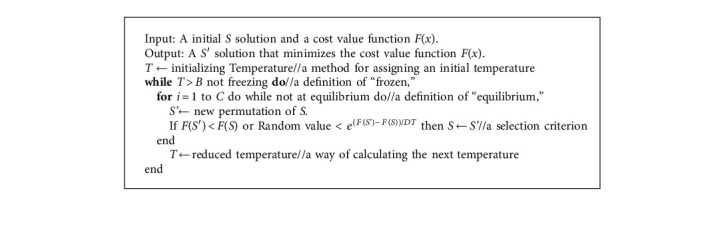
Standard simulated annealing.

**Algorithm 2:**
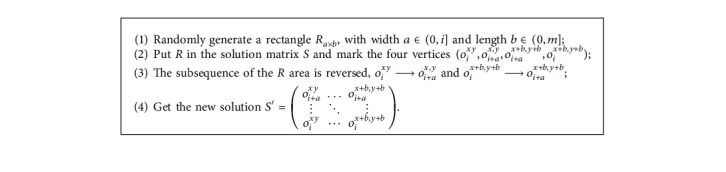
Matrix perturbation.

**Table 1:** Ordnance loading plan.

Aircraft type of ordnance	1	2	3
1	2	1	—
2	1	—	—
3	—	1	—
4	—	—	1

**Table 2:** The SA control factors.

Factors	Levels	Types
Initial solution (A)	3	(A1) randomness (A2) task-based (A3) ascending order
Initial temperature (B)	4	*B*1 = 50, *B*2 = 100, *B*3 = 500, *B*4 = 1000
Cooling rate (C)	4	*C*1 = 0.85, *C*2 = 0.9, *C*3 = 0.95, *C*4 = 0.99
Markov chain (d)	4	*D*1 = 100, *D*2 = 500, *D*3 = 1000, *D*4 = 2000

**Table 3:** Orthogonal array of L16 of our algorithm test.

Experiment	A	B	C	D
1	1	2	3	3
2	2	4	1	2
3	3	4	3	4
4	1	2	1	1
5	1	3	1	4
6	2	1	3	1
7	3	1	1	3
8	2	3	3	2
9	1	1	4	2
10	2	3	2	3
11	3	3	4	1
12	3	1	2	4
13	1	4	2	1
14	2	2	4	4
15	3	2	2	2
16	1	4	4	3

**Table 4:** Results of the orthogonal test.

Factor with level	Completion time	
	Mean S/N ratio	Mean RPD
Random initial (A1)	−73.6779	2.399
Descending initial (A2)	−73.6634	2.216
Ascending initial^*∗*^ (A3)	−73.6504^*∗*^	2.060^*∗*^
*T*0 = 50 (B1)	−73.6656	2.239
*T*0 = 100 (B2)	−73.6638	2.223
*T*0 = 500 (B3)	−73.6669	2.252
*T*0 = 1000^*∗*^ (B4)	−73.6629^*∗*^	2.212^*∗*^
Alpha = 0.85 (C1)	−73.7266	2.962
Alpha = 0.9 (C2)	−73.6791	2.397
Alpha = 0.95 (C3)	−73.6547	2.113
Alpha = 0.99^*∗*^ (C4)	−73.5987^*∗*^	1.453^*∗*^
*L* = 100 (D1)	−73.7602	3.360
*L* = 500 (D2)	−73.6716	2.310
*L* = 1000 (D3)	−73.6344	1.869
*L* = 2000^*∗*^ (D4)	−73.5930^*∗*^	1.387^*∗*^

**Table 5:** Ordnance loading plan.

Ordnance type	Spot task									
	*A*_1_	*A*_2_	*A*_3_	*A*_4_	*A*_5_	*A*_6_	*A*_7_	*A*_8_	*A*_9_	*A*_10_
1	4(2)									
2		4(2)								
3			2(1)	2(1)	2(1)	2(1)	2(1)	2(1)	2(1)	2(1)
4			2(1)	2(1)	2(1)	2(1)	2(1)	2(1)	2(1)	2(1)
5			6(2)	6(2)	6(2)	6(2)				
6							2(2)	2(2)	2(2)	2(2)

**Table 6:** Ordnance assembling time (min).

Ordnance type	1	2	3	4	5	6
Loading time/skid	13	8	7	7	11	6

**Table 7:** Ordnance transport time on flight deck.

Time (s) Upper-stage elevator number	Spot									
	*A*_1_	*A*_2_	*A*_3_	*A*_4_	*A*_5_	*A*_6_	*A*_7_	*A*_8_	*A*_9_	*A*_10_
1	245	199	148	30	61	107	214	213	402	342
2	106	60	190	92	66	67	96	159	345	286
3	381	337	262	166	127	78	74	91	267	208
4	443	398	320	228	189	134	118	80	217	161

**Table 8:** Ordnance loading time (s).

Ordnance type	1	2	3	4	5	6
Time (skid/s)	660	600	480	480	780	360

**Table 9:** Ordnance crew walking time (s).

Spot	Spot									
	*A*_1_	*A*_2_	*A*_3_	*A*_4_	*A*_5_	*A*_6_	*A*_7_	*A*_8_	*A*_9_	*A*_10_
*A*_1_	0	47.53946	168.2	222.6	266.9	331	366	446.9	639	578.8
*A*_2_	47.5	0	142.9	181.2	225.6	289.4	324.9	404.6	596.4	536.1
*A*_3_	168.2	142.9	0	97.8	131.5	189.8	220	302.5	492	432.9
*A*_4_	222.6	181.2	97.8	0	44.4	108.5	143.7	224.3	416.5	356.3
*A*_5_	266.9	225.6	131.5	44.4	0	64.1	99.3	180.1	372.3	312.1
*A*_6_	331	289.4	189.9	108.5	64.1	0	36.1	116	308.2	278.2
*A*_7_	366	324.9	220	143.7	99.3	36.1	0	82.6	274	214.1
*A*_8_	446.9	404.6	302.5	224.3	180.1	116	82.6	0	192.2	132
*A*_9_	639	596.4	492	416.5	372.3	308.1623	274.0073	192.1666	0	60.3
*A*_10_	578.8	536.1	432.9	356.3	312.1	278.2	214.1	132	60.3	0

**Table 10:** Comparison between the ISA and the SA.

Task numbers	Average scheduling time (s)		Average computing time (s)	
	ISA	SA	ISA	SA
15	2965	3100	60	150
30	4200	4500	150	260
45	6100	6500	200	370
60	8000	8600	260	450

## Data Availability

The data used to support the findings of this study are available from the corresponding author upon request.
